# The Efficacy of Probiotics, Prebiotic Inulin-Type Fructans, and Synbiotics in Human Ulcerative Colitis: A Systematic Review and Meta-Analysis

**DOI:** 10.3390/nu11020293

**Published:** 2019-01-30

**Authors:** Erola Astó, Iago Méndez, Sergi Audivert, Andreu Farran-Codina, Jordi Espadaler

**Affiliations:** 1 AB-Biotics, S.A., ESADE Creapolis, Av. Torre Blanca, 57, Sant Cugat del Vallès, E-08172 Barcelona, Spain; asto@ab-biotics.com (E.A.); mendez@ab-biotics.com (I.M.); sergi@ab-biotics.com (S.A.); espadaler@ab-biotics.com (J.E.); 2 Department of Nutrition, Food Science, and Gastronomy, XaRTA – INSA, Faculty of Pharmacy, University of Barcelona, Campus de l’Alimentació de Torribera, Av. Prat de la Riba, 171, Santa Coloma de Gramenet, E-08921 Barcelona, Spain

**Keywords:** ulcerative colitis, remission, probiotic, prebiotic, synbiotic

## Abstract

Studies of probiotics, fructan-type prebiotics, and synbiotics in patients with ulcerative colitis (UC) show significant heterogeneity in methodology and results. Here, we study the efficacy of such interventions and the reasons for the heterogeneity of their results. Eligible random controlled trials were collected from the PUBMED and SCOPUS databases. A total of 18 placebo-controlled and active treatment-controlled (i.e., mesalazine) studies were selected with a Jadad score ≥ 3, including 1491 patients with UC. Data for prebiotics and synbiotics were sparse and consequently these studies were excluded from the meta-analysis. The UC remission efficacy of probiotics was measured in terms of relative risk (RR) and odds ratio (OR). Significant effects were observed in patients with active UC whenever probiotics containing bifidobacteria were used, or when adopting the US Food and Drug Administration (FDA)-recommended scales (UC Disease Activity Index and Disease Activity Index). By the FDA recommended scales, the RR was 1.55 (CI95%: 1.13–2.15, *p*-value = 0.007, *I*^2^ = 29%); for bifidobacteria-containing probiotics, the RR was 1.73 (CI95%: 1.23–2.43, *p*-value = 0.002, *I*^2^ = 35%). No significant effects were observed on the maintenance of remission for placebo-controlled or mesalazine-controlled studies. We conclude that a validated scale is necessary to determine the state of patients with UC. However, probiotics containing bifidobacteria are promising for the treatment of active UC.

## 1. Introduction

The worldwide incidence and prevalence of inflammatory bowel diseases (IBDs) have been increasing over the last few decades [1]. Along with Crohn’s disease (CD), ulcerative colitis (UC) is one of the two major types of IBDs. Unlike CD, which can affect any part of the gastrointestinal tract, from the mouth to the anus, UC characteristically only affects the inner lining of the large bowel [2].

Although the etiology of UC is still unclear, one of the main hypotheses is that it is caused by an excessive immune response to endogenous bacteria in genetically predisposed individuals [3,4]. Therefore, manipulation of the mucosal microbiota to reduce the inflammatory potential of colonizing bacteria is an attractive therapeutic option for UC. Most conventional UC therapies, including the use of compounds containing 5-aminosalicylic acid (5-ASA), corticosteroids, immunosuppressant agents, and anti-tumor necrosis factor (TNF) monoclonal antibodies, suppress intestinal inflammation. However, a subpopulation of patients is refractory to these therapies, or intolerant because of their significant side effects [5]. Also, UC patients can experience frequent relapses. Therefore, treatments that directly modulate the gut microbiota have been studied as adjunctive therapies, or as alternatives to conventional drug therapies [6].

Bacterial microbiota alterations have been well documented in patients with active disease [7]. Metagenomic studies have demonstrated that microbial diversity and intestinal microbiota stability decrease in IBD patients, compared to individuals without IBDs [8]. A recent study identified the microorganisms that invade the terminal ileum and colon of UC-affected individuals as the pro-inflammatory bacteria Enterohemorrhagic *Escherichia coli* (EHEC) and *Campylobacter*, which are both members of the *Enterobacteriaceae* family [8]. A recent meta-analysis identified lower amounts of bacteria from *Clostridium* clusters XIVa and IV in patients with active IBDs [9]. Meanwhile, during active UC, anti-inflammatory *Lactobacillus* and *Pediococcus acidilactici* were absent in fecal samples that were analyzed by fluorescence in situ hybridization. However, with UC in remission, these strains reappeared [7]. Decreased numbers of *Bifidobacterium* spp. were observed in both inflamed UC and CD, while *Lactobacillus* spp. were unchanged during active UC [7]. In addition, a significant decrease in the diversity and richness of the microbiota has been observed in patients with UC in remission, compared with controls, and a further decrease in diversity was observed at relapse [10]. As the composition of the microbiota in UC patients appears to be remarkably unstable [11], there is particular interest in providing such patients with solutions that address their gut dysbiosis to achieve stability, diversity, and optimal abundance of bacterial groups, as observed in healthy controls.

The use of probiotics in IBDs is a potential aid to current conventional therapies. Several studies have focused on the effects of blends of probiotics on enteral microbiota, especially in cases of dysbiosis when the normal concentration of beneficial bacterial flora is reduced due to the presence of pathogenic bacteria [2]. Currently, the standard UC treatment relies on initial managed treatment with corticosteroids and anti-inflammatory agents, such as mesalazine, in conjunction with symptomatic treatment with antidiarrheal agents and rehydration [12].

Probiotics contain viable microorganisms, sufficient amounts of which reach the intestine in an active state for them to exert positive health effects [13]. They mostly include lactic acid-producing bacteria, such as bifidobacteria and lactobacilli; but other organisms, such as *Escherichia coli* and the yeast *Saccharomyces boulardii*, have been reported to have beneficial effects via prolonging remission in patients with IBDs. Although their mechanisms of action have not been established, some studies suggest that these probiotics modulate membrane permeability and the mucosal immune system [6,14]. Among other substances released by bacteria, short-chain fatty acids (SCFAs) have strong immunomodulatory effects and are involved in anti-inflammatory gene regulation processes. Acetic acid, butyric acid, and propionic acid are the most abundant SCFAs [15], and the production of SCFAs seems to play an important role in the maintenance of the gut barrier function [16,17].

Other substances that could potentially be used to treat UC are prebiotics and synbiotics. The International Scientific Association for Probiotics and Prebiotics (ISAPP) consensus statement on the definition and scope of prebiotics states that a prebiotic is “a substrate that is selectively utilized by host microorganisms conferring a health benefit” [18]. It further states, however, that beneficial effects on health must be confirmed in the host that is the target for the intended use and these should always be mediated by the microbiota. Synbiotics are prebiotics combined with probiotic bacteria. Ishikawa (2011) studied the effects of bifidobacterial strains plus GOS synbiotics on patients with UC and concluded that the treatment group significantly improved, showing marked improvements in colonoscopy scores and significant decreases in inflammatory markers [19].

Other meta-analyses of the efficacy of probiotics at treating UC did not analyze subgroups considering the different scales, to determine remission in UC patients [20,21]. This may have led to the identification of a high degree of heterogeneity among studies involving the effects of probiotics, due to the wide variety of scales used to determine UC remission, since patients’ states are observed and scored in different manners according to the differing scales. To avoid this problem of heterogeneity when assessing patients, a study is needed in which the probiotic effect is observed according to the scale that is used to determine the remission state. In addition to the remission scale problems, other meta-analyses have shown that the probiotic VSL#3^®^ (VSL Pharmaceuticals Inc., Ft. Lauderdale, FL, USA) is an effective treatment against active UC [20]. However, it is still unknown whether this is due to the combination of strains presents in the product or the presence of any of its strains or species in particular. Considering that patients with UC exhibit lower concentrations of bifidobacteria than the healthy population, it is possible that their inclusion in VSL#3 makes an effective inducer of remission. Other papers [21,22] report the effects of treatment with bifidobacteria alone or in a mixture, in patients with UC. Thus, the effect of bifidobacteria was another point of interest in our study.

Taking all the foregoing into consideration, in the present systematic review, we consider the efficacy of probiotics as well as inulin-type fructans, prebiotics, and synbiotics with inulin-type fructans in UC patients. Moreover, we performed meta-analysis when the number of studies included allowed this.

## 2. Materials and Methods

This systematic review and the corresponding meta-analysis were performed following the Preferred Reporting Items for Systematic Reviews and Meta-Analyses (PRISMA) guidelines [23].

### 2.1. Data Sources and Searches

The search was performed in PUBMED, SCOPUS, and the Cochrane database, for controlled trials with no restrictions regarding age, sex, year of publication, or the duration of the study. The search terms included “ulcerative colitis” or “inflammatory bowel disease” with the following combined text: “probiotic”, “prebiotic”, “inulin”, “fructo-oligosaccharides”, “FOS”, and “synbiotic” (see full search strategy in Supplementary Data S1). The search focused on human clinical trials referring to treatments with probiotics, prebiotics, or synbiotics in UC patients, and was limited to publications in Spanish or English.

The literature cited in the reviews retrieved was scrutinized to locate additional candidate studies to be included in the systematic review. The titles and abstracts of the papers identified in the initial search were evaluated by two independent reviewers, to ensure their appropriateness for the study.

### 2.2. Study Selection and Data Extraction

Studies duplicated in different databases or searches were included only once. The eligibility of the remaining hits was evaluated by examining titles, abstracts, and full text sequentially. The eligibility criteria were: (1) randomized controlled trials, (2) samples composed of adults or children with active or inactive UC, (3) studies that compared probiotics, prebiotics, or synbiotics with placebos or mesalazine (5-ASA).

Data was extracted by two independent reviewers (EA and AF) onto a Microsoft Excel spreadsheet (Microsoft Corp, Redmond, WA, USA). The data were collected based on study information such as the authors’ names, year of publication, sample size, age of patients, type of medication used, dosage, duration, and outcome. Once the data had been collected, their Jadad score was used to assess the quality of the clinical trials and those with a score of 3 or greater were included in the analyses [24]. In addition, the following clinical data were extracted: the gender of trial participants, the type of probiotic administration, initial states of patients (active or inactive UC), the number of drop-outs and their causes, and the remission scale used. Data were extracted as an intention to-treat (ITT) analysis; patients who did not complete were assumed to be treatment failures (i.e., failed to achieve remission in active UC trials or disease activity relapsed in inactive UC trials) wherever trial reporting allowed for this.

### 2.3. Risk of Bias in Individual Studies

Two investigators (EA and AF) independently evaluated the risk of bias in the studies included using the Cochrane risk-of-bias tool [23]. This evaluates the risk of bias due to random sequence generation, concealment of allocation, blinding, incomplete outcome data, selective reporting, and other sources of bias.

### 2.4. Data Synthesis and Quantitative Synthesis (Meta-Analysis)

The impact of probiotics compared with mesalazine (5-ASA) or a placebo was expressed in terms of RR and OR with 95% confidence intervals (CI95%) in trials of therapy for active UC or no relapse of disease activity in trials of inactive UC. An inverse variance weighting method with random effects modeling was used to compare the RR and OR values between studies. Tables and forest plots with calculated ORs are provided as Supplementary Material, to facilitate comparison with other studies that chose to use ORs as the measure of association.

The heterogeneity between studies was assessed using the χ^2^ test and the *I*^2^ statistic, with a cut off of ≥50% being the definition for a substantial to considerable degree of heterogeneity [25].

The use of subgroup analysis for the meta-analysis was dependent on the clinical score, remission status, study designs, and species of probiotics. More precisely, we performed sensitivity analyses by estimating the RR and ORs with ITT analysis, active and inactive remission of patients, the Ulcerative Colitis Disease Activity Index (UCDAI) and Disease Activity Index (DAI)/Mayo scores, and different probiotics, to evaluate the stability and reduce the heterogeneity of the meta-analysis results. The statistical analysis and calculation of fail-safe numbers [26] were conducted with STATA 14.2 (STATA Corp, College Station, TX, USA) using the command “mar” [27]. Forest plots were generated by Review Manager 5.3 software (The Nordic Cochrane Centre, Copenhagen, Denmark).

## 3. Results

### 3.1. Literature Search and Selected Studies

**Figure 1:**
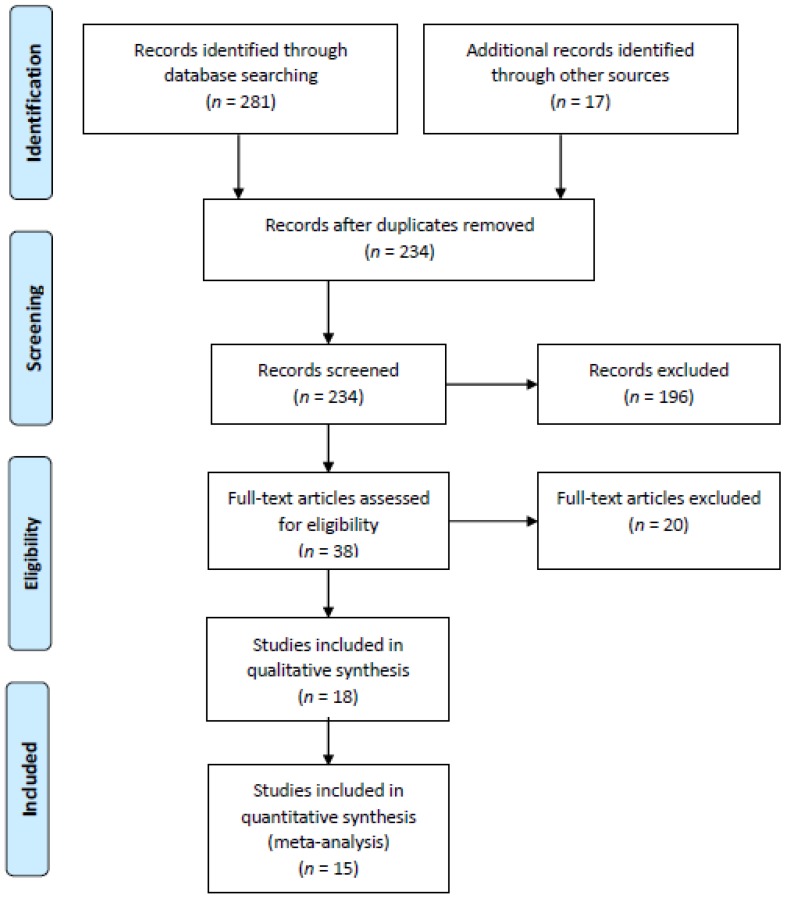
Flow diagram of the selection of studies for systematic review and meta-analysis.

The search strategy generated a total of 298 hits, corresponding to papers published between 1991 and 2018, of which 64 were excluded as repeats (Figure 1). A total of 196 papers were excluded after screening because they reported no probiotic, prebiotic, and synbiotic treatment, or did not focus on a UC clinical trial. The remaining 38 papers were read in full, and 20 were excluded because their Jadad score was lower than 3 [2,28,29,30,31,32,33,34,35,36,37,38,39,40,41,42,43,44,45,46].

We finally analyzed 18 papers [6,47,48,49,50,51,52,53,54,55,56,57,58,59,60,61,62,63], which are summarized in Table 1. Sixteen studied the efficacy of probiotics; while one looked into the efficacy of synbiotics, and one that of prebiotics.

Overall, the studies involved 1491 subjects, with sample sizes ranging from 18 to 327 individuals. Sixteen papers reported data for adults, with a total of 1422 subjects; the other two, data for children and adolescents, with a total of 69 subjects. Moreover, 13 papers focused on active UC; five on inactive UC subjects. However, there were two studies where the initial status of the patients was active and the primary outcome was the induction of remission (delay in outbursts), but in which the maintenance of remission was also studied [57,61]. The papers selected were published between 1997 and 2018; no differences were observed among the percentages of men and women in the studies.

Mutaflor^®^ (Ardeypharm GmbH, Herdecke, Germany) was the most commonly reported treatment, appearing in five papers; followed by VSL#3 which appeared in four papers. The products or probiotics that only appeared in a single paper were *Bifidobacterium longum* 536, Bio-Three^®^ (Toa Pharmaceutical Co., Ltd., Toyama, Japan), *Bifidobacterium infantis* 35624, *Lactobacillus reuteri* ATCC 55730, Probio-Tec AB-25^®^ (Chr. Hansen A/S, Hoersholm Denmark), a composite of *Bifidobacterium breve* Yakult, *Bifidobacterium bifidum* Yakult and *Lactobacillus acidophilus*, and, finally, a mix of *Bifidobacterium breve* Yakult plus *Lactobacillus acidophilus*. There was only one trial that studied a synbiotic product: a mixture of *B. longum* and Synergy 1^®^ (Orafti, Tienen, Belgium). Finally, one paper studied the prebiotic Synergy 1^®^ alone (Table 1).

Outcomes considered in this systematic review were the effects on SCFAs (two papers out of the 18), inflammation levels (five papers), composition of fecal microbiota (three studies) and UC remission (17 papers). We then performed meta-analysis of the papers that studied UC remission with probiotics treatment (15 papers). The three remaining papers were excluded from the meta-analysis because one study did not report the induction or maintenance of UC remission after probiotic treatment [50]; and two studies did not treat patients with probiotics, but with prebiotics [63] or synbiotics [58]. The 15 papers in the meta-analysis include 12 on the efficacy of probiotics in remission, which were placebo-controlled. We differentiated active and inactive patients to study efficacy at inducing or maintaining UC remission. The three remaining papers compared probiotics with mesalazine to study efficacy at maintaining remission.

### 3.2. Risk of Bias and Quality Assessment

The risk of bias was assessed across the 18 studies included (Supplementary Table S1). Evidence of random sequence generation, allocation concealment, and selective reporting was unclear in most studies. As to blinding (participants and personnel), there was a low risk of bias across the included studies, and only one paper was shown to have an unclear risk of bias. No study, except for Matsuoka 2018, showed evidence of maintaining blinding throughout the study, and so (except for Matsuoka 2018) were analyzed as having an unclear risk of bias. The amount of incomplete outcome data was low in most of the studies (10/18), and there was a low risk of other sources of bias in all.

### 3.3. Effect of Probiotics on SCFAs

There were two placebo-controlled trials involving 80 patients that reported the fecal concentrations of SCFAs and the efficacy of probiotics versus placebo [48,59]. One trial reported efficacy in terms of preventing relapse in inactive UC; the other, in terms of inducing remission in active UC cases. In trials on inactive UC patients, the fecal concentration of SCFAs did not differ significantly between the probiotic and placebo groups at any time during treatment. However, the butyrate/acetate ratio at each of the sampling times was significantly higher in fecal samples obtained from patients who had relapsed within six months of fecal collection than in those obtained from patients who remained in remission [48]. In trials with active UC patients, the total SCFA concentration and the butyric and propionic acid concentrations in stools significantly increased after probiotic supplementation [59].

### 3.4. Effect of Probiotics, Prebiotics, and Synbiotics on Gut Inflammation

There were five papers involving 127 patients with active UC that reported the effectiveness of probiotics, prebiotics, or synbiotics versus a placebo on inflammation levels. Systemic inflammatory biomarkers were observed, such as C-reactive protein (CRP) levels, TNF-α, I1-α, IL-6, IL-8, IL-10, and calprotectin, among others. The level of inflammation was assessed in comparison with the control group. All studies reported a significant reduction in inflammation, regardless of whether the study was performed with probiotic, prebiotic, or synbiotic therapy [50,51,55,58,63]. However, two studies assessed probiotics versus placebo treatment in an adult population, but they did not assay the same probiotics [50,55]. Another study assessed probiotics versus placebo treatment, but used a synbiotic therapy based on a *Bifidobacterium longum* probiotic with Synergy 1^®^ prebiotics in adult patients [58]. The results of two studies, one that included prebiotics in an adult population and one in children and young individuals, suggested that probiotics and prebiotics can improve the response to mesalazine by mitigating intestinal inflammation [51,63].

### 3.5. Effect of Probiotics and Synbiotics on Microbiota Composition

Three studies, including a total of 230 patients, studied changes in microbiota composition after probiotic or synbiotic treatment. Matsuoka et al. [47] administered a probiotic to inactive UC patients, and revealed a significant decrease in *Bifidobacterium* species in fecal samples (*p* = 0.006) before relapse, although no effects were detected in the intestinal microbiota. Kato et al. [59] observed that the numbers of fecal *B. breve* and *B. pseudocatenulatum* among bifidobacterial species significantly increased in the probiotic group (*p* < 0.05) but not in the placebo group after administering a probiotic containing bifidobacteria to active UC patients. Other *Bifidobacterium* species and *Bacteroides* species were not significantly changed in either group. Finally, Furrie et al. [58] detected higher numbers of total bifidobacteria on the mucosal surface in active UC patients fed a synbiotic formula that contained *B. longum* and Synergy 1^®^, than in those taking a placebo.

### 3.6. Meta-Analysis of Efficacy of Probiotics at Inducing/Maintaining Remission (Probiotic versus Control)

The meta-analysis finally included 12 placebo-controlled trials, involving a total of 886 individuals. Together, the clinical trials examined the effects of administration (oral or rectal) of probiotics versus placebo or active treatment of UC to induce remission in active UC patients or to maintain remission in inactive UC patients. Detailed study characteristics are provided in Table 1.

Patients were allowed to take concomitant medications such as mesalazine (Supplementary Table S2) in nine out of 12 studies; while in two additional studies all patients took mesalazine in addition to the probiotics or placebo. There was just one study in which patients only took probiotics or a placebo, with no other medication allowed. Matthes et al. [54] compared three different doses of probiotics to a single placebo group. As there were no significant differences between doses on the effect, we performed our analysis using figures calculated by pooling all probiotic doses in one group.

#### 3.6.1. Efficacy of Probiotics in Active UC Patients

**Figure 2:**
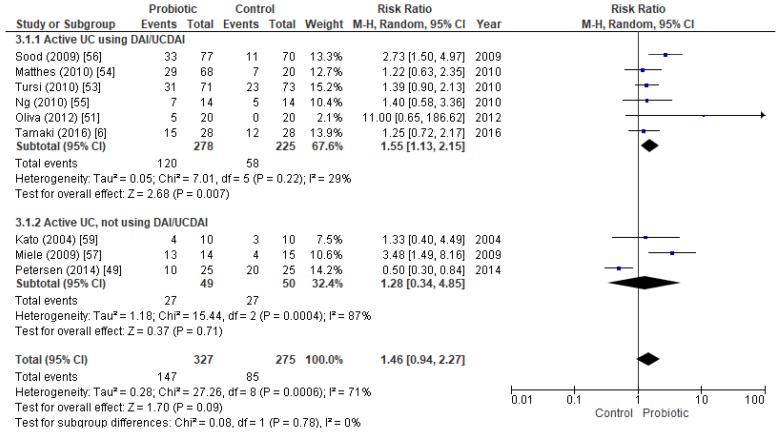
Forest plot of randomized controlled trials assessing remission in active UC patients, assessed with UCDAI or DAI scores (above), or using other scores (below).

Nine papers included 602 active UC patients, and the results showed no significant differences between probiotic and control groups (Figure 2). There was substantial heterogeneity between the studies (*I*^2^ = 71%), and because the *p*-value was 0.09 and the number of studies was sufficiently high, subanalyses were performed to study possible sources of this heterogeneity.

The heterogeneity disappeared when only trials using the UCDAI and DAI indices were included (*I*^2^ = 29%). Meta-analysis of these six trials, totaling 503 individuals, assessing remission with the UCDAI or DAI score found a statistically significant difference between the probiotic and the placebo groups. The RR was 1.55 (CI95% 1.13–2.15, *p*-value = 0.007, *I*^2^ = 29%), and the OR was 2.12 (CI95% 1.36–3.31, *p*-value = 0.000, *I*^2^ = 12%) (Supplementary Figure S1). Other scores (SCCAI, Lichtiger CAI and CAI) were not studied separately as the number of studies that included them was insufficient; but the three studies that did not assess remission with UCDAI or DAI score were analyzed together and the results showed no significant differences between intervention and placebo groups (Figure 2).

Following the method proposed by Rosenthal [26], we estimated the fail-safe number (fsN): the number of studies with non-significant results that would have to be added to change the results of the meta-analysis from significant (*p* < 0.05) to non-significant. The result for the subgroup of studies with active UC patients that used DAI/UCDAI scales was: fsN = 27. As fsN < 5N + 10, where N is the number of case studies in our dataset, we cannot verify the robustness of results against publication bias.

**Figure 3:**
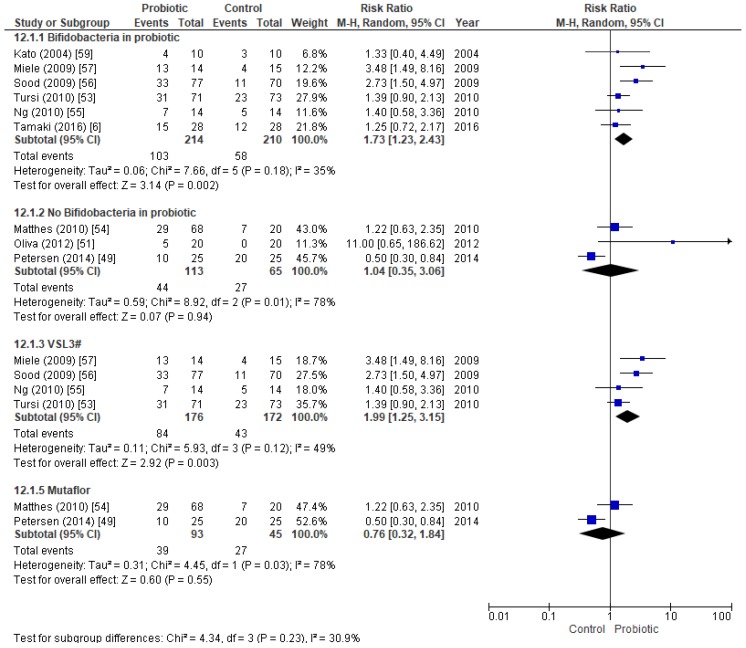
Forest plot of randomized controlled trials in which remission was achieved by active UC patients. Comparison of treatment with VSL#3, Mutaflor, or other probiotics containing bifidobacteria strains as well as those not containing bifidobacteria, all versus a placebo.

Because different probiotics were used to induce remission in active UC patients (Table 1), we also studied the efficacy of Mutaflor, VSL#3, and other probiotics that included *Bifidobacterium* strains, separately (Figure 3). Six studies (424 patients) that used bifidobacteria-containing probiotics were analyzed, and the results suggested that such probiotics were effective in inducing remission in active UC patients. The results were as follows: RR = 1.73 (CI95% 1.23–2.43, *p*-value = 0.002, *I*^2^ = 35%) and OR = 2.50 (CI95% = 1.33–4.70, *p*-value = 0.005, *I*^2^ = 44%) (Supplementary Figure S2). We also calculated fsN for this subgroup and the value obtained (fsN = 36) is less than 5N + 10, so robustness against publication bias is low. Conversely, three studies without bifidobacteria, totaling 168 patients, showed no significant differences between treatment and control groups (Figure 3). VSL#3, when added to conventional therapy at a daily dose of 3.6 × 10^12^ colony-forming unit (CFU/day), effectively induced remission. Four studies (348 patients) that used VSL#3 showed statistically significant differences between the probiotic and the control group, with an RR of 1.99 (CI95% 1.25–3.15, *p*-value = 0.003, *I*^2^ = 49%), and an OR of 3.21 (CI95% 1.31–7.90, *p*-value = 0.01, *I*^2^ = 62%) (Supplementary Figure S2). In this case, fsN = 30 indicating a low robustness against publication bias. Finally, two aggregated Mutaflor studies (138 patients) could not demonstrate that this treatment promotes remission in active UC patients (Figure 3).

#### 3.6.2. Efficacy of Probiotics in Inactive UC Patients

**Figure 4:**
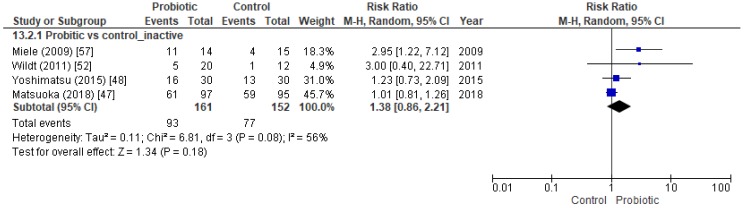
Forest plot of randomized controlled trials focused on the maintenance of remission in inactive UC patients.

Four trials that included 313 inactive UC patients attempted to determine the efficacy of probiotics at maintaining remission, but no statistically significant differences were detected between the probiotic and control groups (Figure 4) (Supplementary Figure S3). One of these papers studied the remission rate in active UC patients, but also assessed the maintenance of remission until the end of the study [57].

### 3.7. Efficacy of Probiotics at Maintaining Remission (Probiotics versus Mesalazine)

**Figure 5:**
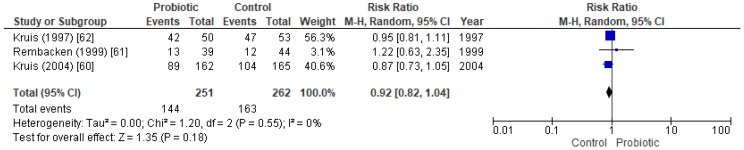
Forest plot of randomized controlled trials regarding the maintenance of remission in inactive UC patients when comparing the probiotic Mutaflor with mesalazine.

To compare probiotics against mesalazine in maintaining remission in UC patients, the meta-analysis included three papers involving a total of 513 individuals. One of these papers studied both the remission rate in active UC patients and the maintenance of remission at follow-up, and it was the only study that permitted concomitant medication (Supplementary Table S2) [61]. Together, the clinical trials examined the effect of oral administration of the probiotic Mutaflor against mesalazine, at maintaining remission in inactive UC patients. However, the results showed no significant differences between probiotic and control groups, suggesting that the probiotic was equally as effective as mesalazine at maintaining UC remission (Figure 5) (Supplementary Figure S4).

## 4. Discussion

Guidelines and recommendations from scientific experts and institutions support the safety of probiotics, and state that some are at least as effective as conventional therapy at achieving remission in UC patients in both adult and pediatric populations [13]. Therapy with probiotics, prebiotics, and synbiotics has been proposed to increase the production of SCFAs [18]. SCFAs, in combination with bifidobacteria, are involved in the reduction of inflammation [18,64], and are recognized as having mechanistic links to health outcomes [18,65].

Our analysis also suggests that probiotics effectively help to achieve remission in active UC cases, as this effect seems to be statistically significant. When a subanalysis was performed only on the papers that compared probiotics against a placebo and patient remission was determined with only the UCDAI and DAI scales, the lowest heterogeneity among studies was achieved.

Both the UCDAI and DAI (total score of 0 to 12) incorporate scores for stool frequency, rectal bleeding, endoscopic findings, and clinical assessment of disease activity [66]. Conversely, CAI (0–23), SCCAI (0–20), and Lichtiger CAI (0–21) also include more general items, such as general well-being, extra-intestinal features, fever, anemia, and the erythrocyte sedimentation rate (ESR) [66]. Thus, these latter scales include more general extra-intestinal features, where probiotics would seem less likely to have an effect. Moreover, laboratory markers, which are more objective, cannot be replaced by the other parameters [67], and signs and symptoms are best assessed by a patient-reported outcome instrument, as opposed to clinician-reported outcome instruments [68]. Furthermore, the treatment effect was statistically significant in studies that used the UCDAI and DAI scales, but not in those that used other scales, with heterogeneity considerably higher in the latter case. This may mean that the use of the same method to determine remission is an aid in understanding the effects of probiotics in patients with UC.

The most commonly used disease activity scales among the trials in this study were the UCDAI and the DAI. In our meta-analysis, we considered that both scales were similar enough to be considered to measure the same construct in different studies [69]. Although significant results were observed when using studies that identified remission with UCDAI or DAI, there is not yet a validated scale for determining remission in patients with UC [70]. Thus, the use of either the UCDAI or DAI scale, instead of other scales, cannot be justified based on these observations.

Nonetheless, probiotics containing bifidobacteria were demonstrated to promote remission in patients with active UC, while probiotics not containing bifidobacteria did not have a beneficial effect. Heterogeneity was not observed among the bifidobacteria studies, but was greater than that observed in the meta-analysis of trials that determined remission with the UCDAI and DAI scales. Duranti et al. (2016) [71] suggest that patients with UC have reduced levels of bifidobacteria (as assessed by 16S ribosomal RNA (rRNA) compared to the healthy population. It, therefore, seems logical that the use of a probiotic treatment containing bifidobacteria would improve patient health; and this suggests potential therapeutic roles for this genus and probiotics containing these bacteria.

VSL#3 was shown to be an effective probiotic for achieving remission in active UC patients. It has previously been shown that VSL#3 is effective in UC and that it is capable of controlling inflammation, and lengthening the remission period [72]. Although our meta-analysis includes a limited number of studies showing the efficacy of the probiotic VSL#3, administration was found to be effective in patients with active UC. Other authors report similar results, and have showed that VSL#3 can effectively induce and maintain remission in UC patients [21,22,73]. The efficacy of VSL#3 could be due to the variety of bacteria it contains, or specifically to the presence of one bifidobacteria strain. In our meta-analysis, the reduction in heterogeneity between studies was higher when treatments with probiotics that contained bifidobacteria were compared to studies using VSL#3. Shen et al. 2014 [22], in their meta-analysis, found no beneficial effects of probiotics with bifidobacteria; but unlike us, they included the Furrie et al. 2005 [58] study, where the treatment was synbiotic. Additionally, they did not include as many studies; for example, they excluded studies that performed VSL#3 treatments. In our meta-analysis, we did not include studies with synbiotics, because, in such studies, it is not fully known whether the effect is due to the probiotic or prebiotic.

A previous systematic review and meta-analysis of six trials evaluated the EcN 1917 probiotic against mesalazine treatment, and determined that EcN is equivalent to mesalazine in preventing disease relapse [73]. Our study suggests similar effects of the EcN 1917 probiotic on maintaining remission as those of mesalazine treatment (RR = 0.92, CI95% 0.82–1.04, *p*-value = 0.18). The difference in the number of studies was due to the fact that our subanalysis only included probiotics versus placebo studies. This implied that two studies were excluded because they compared a probiotic-plus-mesalazine to placebo-plus-mesalazine. Additionally, one study included in that review [73] was also excluded from ours because it obtained a Jadad scale score < 3.

Our systematic review has revealed a scarcity of reliable data for prebiotics and synbiotics. There were not enough studies to perform meta-analysis of the effect of prebiotics or synbiotics, so the decision was made to exclude these from our meta-analysis. They are not included in our prebiotics meta-analysis because they are different treatments.

While the results of the present review and meta-analysis suggest a beneficial effect of probiotic treatment in active UC patients, there were some limitations when comparing the different studies. The clinical trials differed in terms of their inclusion and exclusion criteria, regarding, for example, treatment, concomitant medications, diversity in the probiotic strains used, and the probiotic schedules and concentrations. Moreover, most of the studies included had few participants, some had many drop-outs and, most importantly, a variety of scales was used to define remission status.

Although there is no ideal or validated scale to define remission status, the FDA has indicated that while this is the case, the UCDAI and DAI are the most commonly used scales and are recommended [66,68]. In addition, a systematic review containing 153 trials, of which 116 used the UCDAI scale, evaluated the definition and evidence for remission endpoints in UC from the point of view of two particular scales (UCEIS and UCDAI), and recommended that an international consensus of remission should be sought as a matter of urgency before establishing a gold standard for outcome measurement. This would lead towards standardization of clinical trial protocols and advance in patient care [74]. Thus, presently, it is difficult to determine the best possible outcomes due to a lack of homogeneity of the clinical trial protocols.

Therefore, we still do not know the necessary doses or the duration of the probiotic treatments that are needed to obtain an optimal effect. It could be that by using the same scale to define the remission state, beneficial remission effects occur in patients with UC treated with more commercial probiotic strains. Thus, we suggest that more studies involving prebiotics or synbiotics are needed. A few studies have shown that inulin increases bifidobacteria levels, having a beneficial effect on the gut [75]. Thus, this genus could play an important therapeutic role. However, further studies are needed to determine the effects of probiotics on SCFA production, gut inflammation, and microbiota composition. These factors should also be studied for prebiotics and synbiotics, in order to gauge their effects in UC treatment. Other interesting alternatives for the treatment of ulcerative colitis, such as fecal microbiota transplantation (FMT), are currently being studied [76]. A Cochrane systematic review suggests that FMT may increase the proportion of UC patients achieving clinical remission [77]. However, not many controlled or randomized studies have been published using FMT for indications other than *C. difficile* infection, so more studies are also needed before recommending FMT for the treatment of ulcerative colitis.

Finally, the definition of the term “probiotic” is very generic and lacks specificity; and we must stress the importance of adequate doses and the determination of probiotics or strains that cause beneficial effects. Larger sample sizes and dose-finding randomized controlled clinical trials are probably needed to clarify the role of probiotics in the treatment of UC.

## 5. Conclusions

In conclusion, our meta-analysis shows that the use of probiotics seems to be beneficial in terms of reaching remission in patients with UC, but this observation depends strongly on the UC scale adopted. More precisely, the significant effect is only observed with the UCDAI and DAI scales, which are those recommended by the FDA for UC clinical trials with pharmaceuticals; not with other scales. Probiotic composition also seems to play a relevant role, with bifidobacteria-containing probiotics seemingly being most beneficial.

## Figures and Tables

**Table 1:** Main characteristics of randomized controlled trials of probiotics versus placebo or mesalazine in inducing or maintaining remission.

Study	Sample/Initial State	Age	Ulcerative Colitis Remission Score	Comparison	Therapy Used	Control Used
* Matsuoka et al. 2018 [47]	192/Inactive	≥18	DAI	Probiotic vs. control ^a^	*B. breve* Yakult and *L. acidophilus* ^b^	Placebo
* Tamaki et al. 2016 [6]	56/Active	≥18	UCDAI	Probiotic vs. control ^a^	BB536 ^b^	Placebo
* Yoshimatsu et al. 2015 [48]	60/Inactive	≥18	UCDAI	Probiotic vs. control ^a^	Bio-Three	Placebo
* Petersen et al. 2014 [49]	50/Active	≥18	CAI	Probiotic vs. control ^a^	Mutaflor	Placebo
Groeger et al. 2013 [50]	22/Active	≥18	Not assessed	Probiotic vs. control	*B. infantis* 35624 ^b^	Placebo
* Oliva et al. 2012 [51]	40/Active	6–18	DAI	Probiotic vs. control (both with mesalazine)	*L. reuteri* ATCC 55730	Placebo
* Wildt et al. 2011 [52]	32/Inactive	≥18	SCCAI	Probiotic vs. control	Probio-Tec AB-25 ^b^	Placebo
* Tursi et al. 2010 [53]	144/Active	≥18	UCDAI	Probiotic vs. control ^a^	VSL#3 ^b^	Placebo
* Matthes et al. 2010 [54]	88/Active	≥18	DAI	Probiotic vs. control ^a^	Mutaflor	Placebo
* Ng et al. 2010 [55]	28/Active	≥18	UCDAI	Probiotic vs. control ^a^	VSL#3 ^b^	Placebo
* Sood et al. 2009 [56]	147/Active	≥18	UCDAI	Probiotic vs. control ^a^	VSL#3 ^b^	Placebo
* Miele et al. 2009 [57]	29/Active	8–16	Lichtiger CAI	Probiotic vs. control (both with mesalazine)	VSL#3 ^b^	Placebo
Casellas et al. 2007 [63]	19/Active	≥18	CAI	Prebiotic vs. control (both with mesalazine)	Synergy 1	Placebo
Furrie et al. 2005 [58]	18/Active	≥18	CAI	Synbiotic vs. control	*B. longum*/Synergy1 ^b^	Placebo
* Kato et al. 2004 [59]	20/Active	≥18	CAI	Probiotic vs. control ^a^	*B. breve* Yakult, *B. bifidum* Yakult and *L. acidophilus* ^b^	Placebo
* Kruis et al. 2004 [60]	327/Inactive	≥18	CAI	Probiotic vs. mesalazine	Mutaflor	Mesalazine
* Rembacken et al. 1999 [61]	116/Active	≥18	CAI	Probiotic vs. mesalazine	Mutaflor	Mesalazine
* Kruis et al. 1997 [62]	103/Inactive	≥18	CAI	Probiotic vs. mesalazine	Mutaflor	Mesalazine

UCDAI: ulcerative colitis disease activity index; DAI: disease activity index; CAI: clinical activity index; SCCAI: simple clinical colitis activity index. * Studies included in the meta-analysis; ^a^ Therapy and Control allowed concomitant medication; ^b^ Probiotics that contain *Bifidobacterium* strains.

## Supplementary Materials

The following are available online at http://www.mdpi.com/2072-6643/11/2/293/s1, Data S1: Search strategy, Table S1: Risk of bias in selected studies, Table S2: Concomitant medication in the studies, Figure S1: Forest plot with ORs of randomized controlled trials assessing remission in active UC patients, assessed with UCDAI or DAI scales, or using other scales, Figure S2: Forest plot with ORs of randomized controlled trials of remission in active UC patients, when comparing VSL#3, Mutaflor, or other probiotics containing *Bifidobacterium* strains or not, all versus placebo, Figure S3: Forest plot with ORs of randomized controlled trials of maintaining remission in inactive UC patients, Figure S4: Forest plot with ORs of randomized controlled trials in maintaining remission in inactive UC patients when comparing the probiotic Mutaflor with mesalazine.
