# Supplementary material for: The Efficacy of Probiotics, Prebiotic Inulin-Type Fructans, and Synbiotics in Human Ulcerative Colitis: A Systematic Review and Meta-Analysis
Source: Nutrients. 2019 Jan 30;11(2):293. doi: 10.3390/nu11020293 (PMC6412539; doi:10.3390/nu11020293)

nutrients

MDPI

Supplementary Materials and Data of Review:

The Efficacy of Probiotics, Prebiotic Inulin-Type Fructans, and Synbiotics in Human Ulcerative Colitis: A Systematic Review and Meta-Analysis

Supplementary Data S1 Search strategy

Only clinical trial publications and human filters were used in the literature search performed in PUBMED. The studies were searched using the terms “ulcerative colitis” in combination with “probiotic”, “synbiotic”, and “inflammatory bowel disease” in combination with “prebiotic”, “inulin”, “FOS” and “fructo-oligosaccharides”.

The search in SCOPUS was performed using combinations of the terms “ulcerative colitis” AND “inulin” limited to document types “Article”. Another search used “ulcerative colitis” AND “probiotic” AND “human” limited to document type “Article” and the subareas “Medicine”, “Immunology and Microbiology” and “Health Professions” and the exact key words “Controlled Study”, “Clinical Article”, “Clinical Trials”, “Controlled Clinical Trial” and “Double-Blind Method”. An additional search using the combinations “ulcerative colitis” AND “synbiotic” AND “human” limited to document type “Article” and excluding the exact key word “Crohn Disease”. In all cases, the search was limited to the languages “Spanish” and “English”

Supplementary Table S1 Risk of bias of selected studies

	Random Sequence generation	Allocation concealment	Blinding (participants and personnel)	Blinding (outcome assessment)	Incomplete outcome data	Selective reporting	Other sources of bias
Matsuoka et al. 2018	?	+	+	+	+	+	+
Tamaki et al. 2016	?	+	+	?	-	?	+
Yoshimatsu et al. 2015	?	?	+	?	-	+	+
Petersen et al. 2014	?	+	+	?	+	+	+
Groeger et al. 2013	-	-	+	?	-	?	+
Oliva et al. 2012	+	+	+	?	-	+	+
Wildt et al. 2011	?	+	+	?	+	+	+
Tursi et al. 2010	?	+	+	?	+	?	+
Matthes et al. 2010	?	+	+	?	-	+	+
Ng et al. 2010	?	-	+	?	+	?	+
Sood et al. 2009	+	+	+	?	-	?	+
Miele et al. 2009	+	+	+	?	-	?	+
Furrie et al. 2005	?	+	+	?	-	-	+
Kato et al. 2004	+	?	?	?	+	?	+
Kruis et al. 2004	?	?	+	?	+	?	+
Rembacken et al. 1999	?	?	+	?	-	?	+
Kruis et al. 1997	?	?	+	?	-	?	+
Casellas et al. 2007	?	?	+	?	+	?	+

LOW	+
UNCLEAR	?
HIGH	-

Supplementary Table S2 Concomitant medications used in the studies

		Concomitant medication for ulcerative colitis permitted during the study:
Matsouka et al.	2018 July	1) Mesalazine (Pentasa®, Salazopyrin®, Asacol®) 96 patients in probiotic group and 94 in placebo group; 2) drugs for intestinal disorder; 3) loperamide hydrochloride 4) drugs for diarrhea-predominant irritable bowel syndrome (Colonel®Cerekinon®, Irribow®, Phelloberin®)
Tamaki et al.	2016 Jan	1) Mesalazine (probiotic group: 25 patients; placebo group: 28 patients); 2) prednisolone (probiotic group: 6 patients; placebo group: 11 patients); 3) azathioprine (probiotic group: 5 patients; placebo group: 9 patients)
Yoshimatsu et al.	2015 May 21	1) Mesalazine (Pentasa®: probiotic group: 11 patients; placebo group: 13 patients); 2) salazosulfapyridine (Salazopyrin®: probiotic group: 10 patients; placebo group: 9 patients); 3) Pentasa + Salazopyrin (probiotic group: 1 patient; placebo group: 0 patients)
Petersen et al.	2014 Nov	1) Systemic mesalazine (16 patients in probiotic group; 15 patients in placebo group; 2) topical Mesalazine (13 patients in probiotic group; 18 patients in placebo group); 3) azathioprine/6-mercaptopurine (2 patients in probiotic group; 6 patients in placebo group); 4) topical prednisolone (one patient in each group)
Oliva et al.	2012 Feb	1) Oral mesalazine (all patients)
Wildt et al.	2011 Apr	Not permitted
Tursi et al.	2010 Oct	1) Mesalazine (65 patients in probiotic group; 69 patients in placebo group); 2) Balsalazide (2 patients in probiotic group; 2 patients in placebo group); 3) azathioprine (only one in probiotic group; 4) methotrexate (only one in probiotic group). There were 2 patients in each group (probiotic and placebo) that received mesalazine + azathioprine
Matthes et al.	2010 Apr 15	1) Antidiarrhoeal, anti-inflammatory (mesalazine was the most common) and/or anti-infective medication (53 patients of 88 total patients); 2) corticoids (14 patients of 88 total patients)
Ng et al.	2010 Aug	1) Mesalazine (5 patients in probiotic group; 9 patients in placebo group); 2) mesalazine+ 6-mercaptopurine (2 patients in probiotic group; 3 patients in placebo group); 7 patients in probiotic group and 2 patients in placebo group did not receive concomitant medication
Sood et al.	2009 Nov	1) Mesalazine (69 patients in probiotic group; 47 patients in placebo group) 2) mesalazine + immunosuppressants (5 patients in probiotic

		group; 15 patients in placebo group); 3) immunosuppressants (one patient in each group); the immunosuppressants used were azathioprine or 6-mercaptopurine
Miele et al.	2009 Feb	1) Mesalazine (all patients); 2) steroids (all patients)
Kato et al.	2004 Nov	1) Mesalazine (9 patients in probiotic group; 9 patients in placebo group); 2) salazosulphapyridine (SASP) (one patient in each group)
Kruis et al.	2004 Nov	Not permitted
Rembacken et al.	1999 Aug 21	1) Hydrocortisone; 2) prednisolone
Kruis et al.	1997 Oct	Not permitted

Supplementary Figure S1. Forest Plot with Odds Ratios (ORs) of randomized controlled trials assessing remission in active UC patients assessed with UCDAI or DAI scores (above) or using other scores (below).

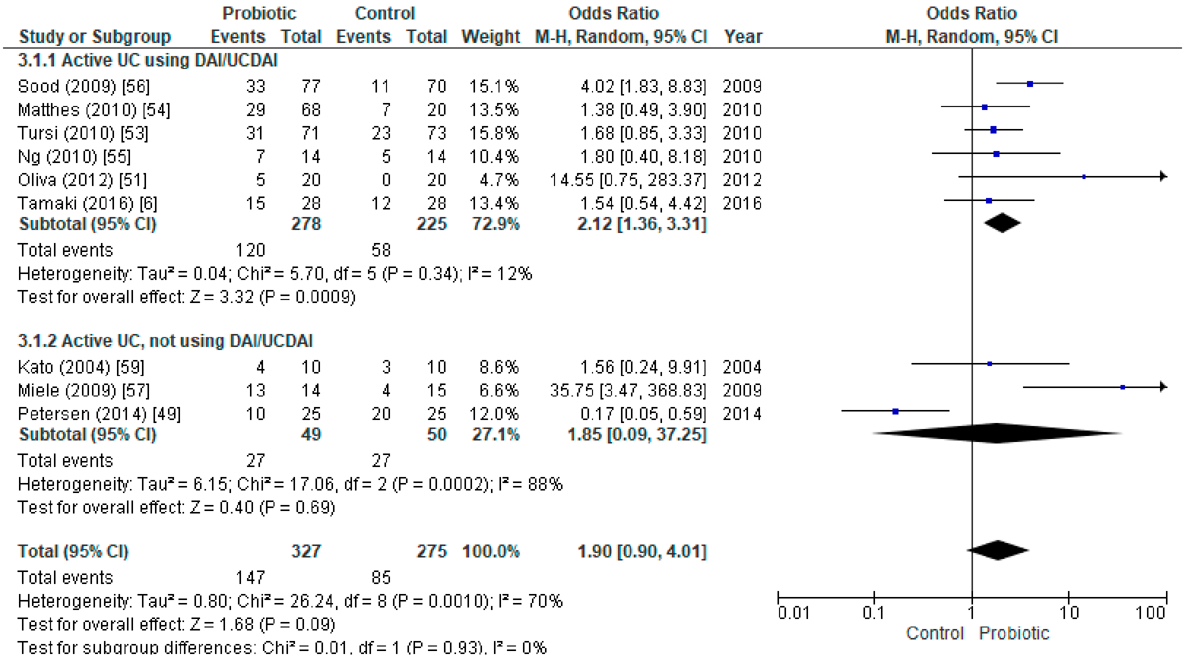

Supplementary Figure S2. Forest Plot with ORs of randomized controlled trials regarding the achievement of remission in active UC patients when compared probiotics containing Bifidobacteria strains, VSL3#, or Mutaflor, or those not containing Bifidobacteria versus a placebo.

Study or Subgroup	Probiotic Events	Probiotic Total	Control Events	Control Total	Weight	Odds Ratio M-H, Random, 95% CI	Year
**12.1.1 Bifidobacteria in probiotic**							
Kato (2004) [59]	4	10	3	10	9.1%	1.56 [0.24, 9.91]	2004
Miele (2009) [57]	13	14	4	15	6.2%	35.75 [3.47, 368.83]	2009
Sood (2009) [56]	33	77	11	70	25.2%	4.02 [1.83, 8.83]	2009
Tursi (2010) [53]	31	71	23	73	28.0%	1.68 [0.85, 3.33]	2010
Ng (2010) [55]	7	14	5	14	12.3%	1.80 [0.40, 8.18]	2010
Tamaki (2016) [6]	15	28	12	28	19.2%	1.54 [0.54, 4.42]	2016
**Subtotal (95% CI)**		**214**		**210**	**100.0%**	**2.50 [1.33, 4.70]**	
Total events	103		58				
Heterogeneity: Tau² = 0.25; Chi² = 8.93, df = 5 (P = 0.11); I² = 44%							
Test for overall effect: Z = 2.84 (P = 0.005)							
**12.1.2 No Bifidobacteria in probiotic**							
Matthes (2010) [54]	29	68	7	20	39.5%	1.38 [0.49, 3.90]	2010
Oliva (2012) [51]	5	20	0	20	22.9%	14.55 [0.75, 283.37]	2012
Petersen (2014) [49]	10	25	20	25	37.6%	0.17 [0.05, 0.59]	2014
**Subtotal (95% CI)**		**113**		**65**	**100.0%**	**1.07 [0.14, 8.35]**	
Total events	44		27				
Heterogeneity: Tau² = 2.51; Chi² = 10.74, df = 2 (P = 0.005); I² = 81%							
Test for overall effect: Z = 0.06 (P = 0.95)							
**12.1.3 VSL3#**							
Miele (2009) [57]	13	14	4	15	11.2%	35.75 [3.47, 368.83]	2009
Sood (2009) [56]	33	77	11	70	33.4%	4.02 [1.83, 8.83]	2009
Ng (2010) [55]	7	14	5	14	19.8%	1.80 [0.40, 8.18]	2010
Tursi (2010) [53]	31	71	23	73	35.7%	1.68 [0.85, 3.33]	2010
**Subtotal (95% CI)**		**176**		**172**	**100.0%**	**3.21 [1.31, 7.90]**	
Total events	84		43				
Heterogeneity: Tau² = 0.47; Chi² = 7.87, df = 3 (P = 0.05); I² = 62%							
Test for overall effect: Z = 2.54 (P = 0.01)							
**12.1.5 Mutaflor**							
Matthes (2010) [54]	29	68	7	20	51.5%	1.38 [0.49, 3.90]	2010
Petersen (2014) [49]	10	25	20	25	48.5%	0.17 [0.05, 0.59]	2014
**Subtotal (95% CI)**		**93**		**45**	**100.0%**	**0.50 [0.06, 3.93]**	
Total events	39		27				
Heterogeneity: Tau² = 1.89; Chi² = 6.42, df = 1 (P = 0.01); I² = 84%							
Test for overall effect: Z = 0.66 (P = 0.51)							

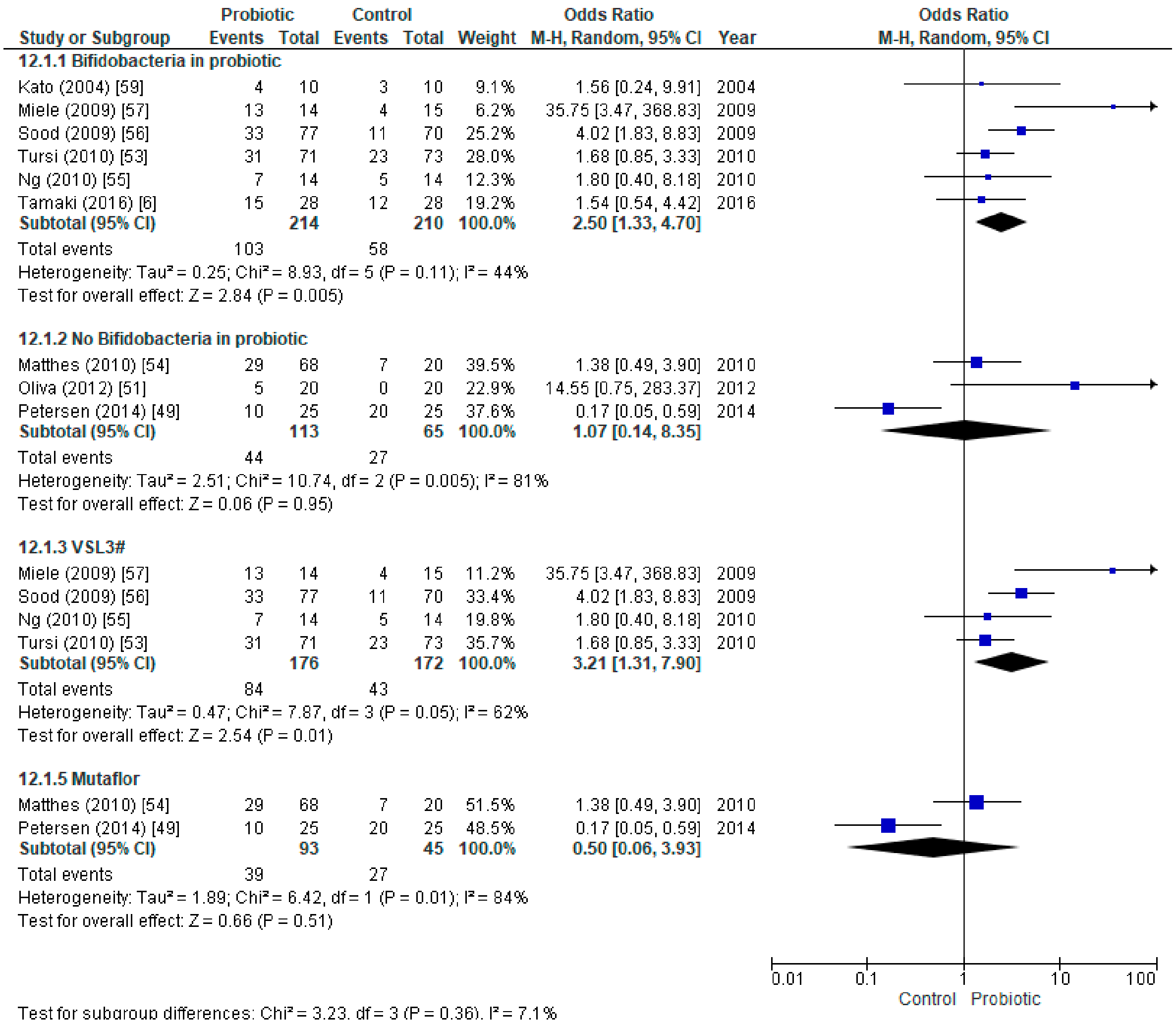

Test for subgroup differences: Chi² = 3.23, df = 3 (P = 0.36), I² = 7.1%

Supplementary Figure S3. Forest Plot with ORs of randomized controlled trials regarding the maintenance of remission in inactive UC patients

Study or Subgroup	Probiotic Events	Probiotic Total	Control Events	Control Total	Weight	Odds Ratio M-H, Random, 95% CI	Year
**13.2.1 Probitic vs control_inactive**							
Miele (2009) [57]	11	14	4	15	17.8%	10.08 [1.82, 56.00]	2009
Wildt (2011) [52]	5	20	1	12	11.9%	3.67 [0.37, 35.98]	2011
Yoshimatsu (2015) [48]	16	30	13	30	30.1%	1.49 [0.54, 4.14]	2015
Matsuoka (2018) [47]	61	97	59	95	40.2%	1.03 [0.58, 1.85]	2018
**Subtotal (95% CI)**		**161**		**152**	**100.0%**	**2.01 [0.81, 5.00]**	
Total events	93		77				
Heterogeneity: Tau² = 0.45; Chi² = 6.85, df = 3 (P = 0.08); I² = 56%							
Test for overall effect: Z = 1.51 (P = 0.13)							

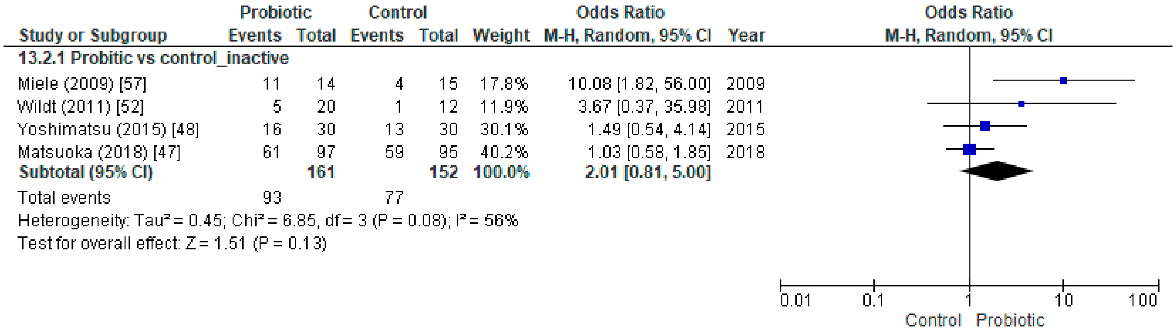

Supplementary Figure S4. Forest Plot with ORs of randomized controlled trials regarding the maintenance of remission in inactive UC patients as compared with the probiotic Mutaflor with mesalazine.

	Probiotic		Control			Odds Ratio		Odds Ratio
**Study or Subgroup**	**Events**	**Total**	**Events**	**Total**	**Weight**	**M-H, Random, 95% CI**	**Year**	**M-H, Random, 95% CI**
Kruis (1997) [62]	42	50	47	53	11.0%	0.67 [0.21, 2.09]	1997	
Rembacken (1999) [61]	13	39	12	44	16.1%	1.33 [0.52, 3.41]	1999	
Kruis (2004) [60]	89	162	104	165	72.8%	0.72 [0.46, 1.11]	2004	
**Total (95% CI)**		**251**		**262**	**100.0%**	**0.79 [0.54, 1.15]**		
Total events	144		163					

Heterogeneity: Tau² = 0.00; Chi² = 1.47, df = 2 (P = 0.48); I² = 0%

Test for overall effect: Z = 1.26 (P = 0.21)

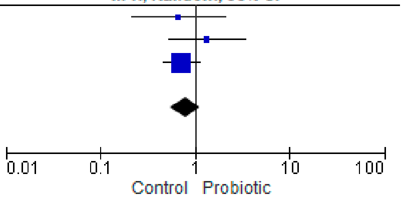

Supplement: Supplementary file 1 [file nutrients-11-00293-s001.pdf]
